# Crystal structure of 2-[2-(pyridin-3-yl)diazen-1-yl]aniline

**DOI:** 10.1107/S2056989018008605

**Published:** 2018-06-26

**Authors:** Morten K. Peters, Christian Näther, Rainer Herges

**Affiliations:** a Otto-Diels-Institut für Anorganische Chemie, Christian-Albrechts-Universität Kiel, Otto-Hahn-Platz 4, D-24098 Kiel, Germany; b Institut für Anorganische Chemie, Christian-Albrechts-Universität Kiel, Max-Eyth Str. 2, D-24118 Kiel, Germany

**Keywords:** crystal structure, hydrogen bonding, azo­pyridine

## Abstract

The crystal structure of the title compound, C_11_H_10_N_4_, comprises mol­ecules in a *trans* conformation for which all the atoms are located in general positions. The six-membered rings are coplanar and this arrangement might be stabilized by intra­molecular N—H⋯N hydrogen bonding. In the crystal, the mol­ecules are linked into helical chains parallel to the *b* axis *via* N—H⋯N hydrogen bonding. The mol­ecular packing shows a herringbone-like pattern along the *a* axis. Comparison of the X-ray powder diffraction with that calculated from single crystal data proves that a pure crystalline phase was obtained and UV–Vis measurements reveal that only the *trans* isomer is present.

## Abstract 2

In the crystal structure of the title compound the mol­ecules are coplanar and are linked into helical chains *via* N—H⋯N hydrogen bonding between one of the amino H atoms and the pyridine N atoms.

## Chemical context   

Azo­benzenes are among the most frequently used photochromic compounds with numerous applications in different fields being reported (Szymański *et al.*, 2013 ▸; Merino & Ribagorda, 2012 ▸; Kay *et al.*, 2007 ▸). Moreover, azo­benzenes are easily accessible, and their photochromic functions are quite reliable. The stretched *trans* isomer is usually the thermodynamically stable conformation. Upon irradiation with UV light, the bent *cis* isomer is formed. This *cis* conformation switches back to the *trans* isomer either upon irradiation with visible light or thermochemically (Hartley, 1937 ▸). A highly important variation of azo­benzenes are azo­pyridines, as pyridines coordinate to various metals, *e.g*. nickel (Thies *et al.*, 2010 ▸; Dommaschk *et al.*, 2015*c* ▸). Thus, azo­pyridines can be used as switchable ligands. In this context, we have reported an approach to switch the spin state of azo­pyridine-function­alized Ni-porphyrins (Thies *et al.*, 2011 ▸, 2012 ▸; Venkataramani *et al.*, 2011 ▸; Dommaschk *et al.*, 2015*a* ▸,*b* ▸). Aiming at further functionalization of azo­pyridines and in view of applications as mol­ecular spin switches, we have synthesized 2-[2-(pyridin-3-yl)diazen-1-yl]aniline and report here its mol­ecular and crystal structure.
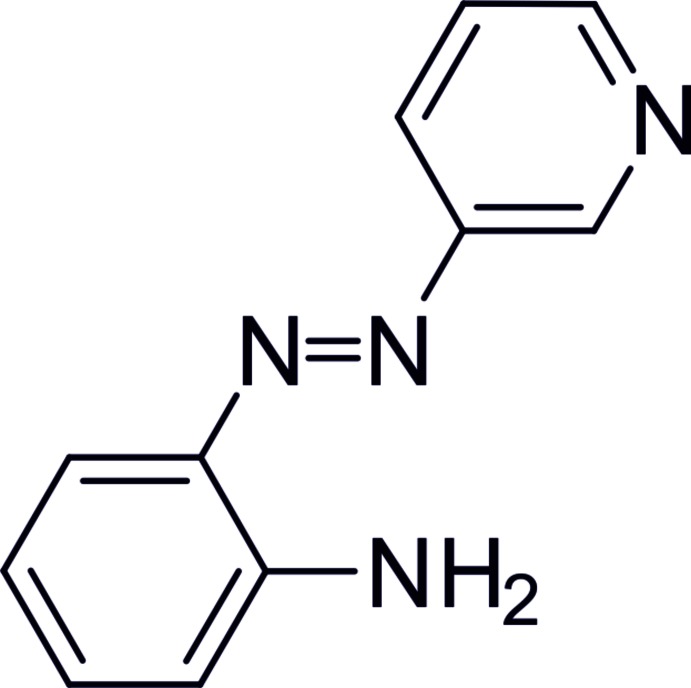


## Structural commentary   

**Figure 1:**
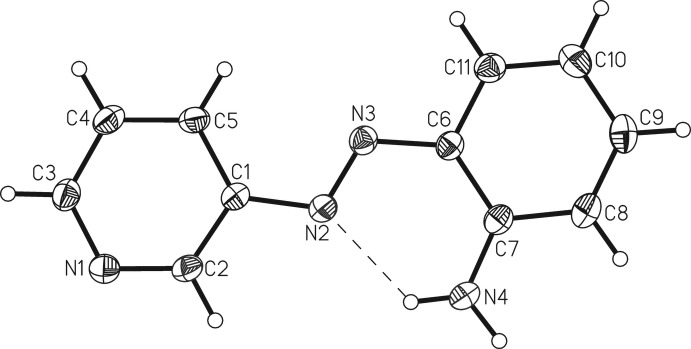
Mol­ecular structure of the title compound with labeling and displacement elliposids drawn at the 50% probability level. The intra­molecular N—H⋯N hydrogen bond is shown with dashed lines.

The crystal structure of the title compound comprises 2-[2-(pyridin-3-yl)diazen-1-yl]aniline mol­ecules, located on general positions, adopting a *trans*-conformation with a C1—N2—N3—C6 torsion angle of −179.80 (8)°, which corresponds to the energetically favored arrangement (Fig. 1 ▸). The two six-membered rings are coplanar [the maximum deviation from the least-squares plane for all non-H atoms is 0.0569 (9) Å for N4] and the dihedral angle between the ring planes is 0.11 (8)°. The amino H atoms are also in the plane of the adjacent benzene ring. There is an intra­molecular N—H⋯N hydrogen bond between one of the amino H atoms and one nitro­gen atom of the azo group with an N⋯H distance of 2.066 (15) Å and an N—H⋯N angle of 127.30 (12)° (Table 1 ▸). Even if this corresponds to a weak inter­action, it might stabilize the planar arrangement.

## Supra­molecular features   

**Figure 2:**
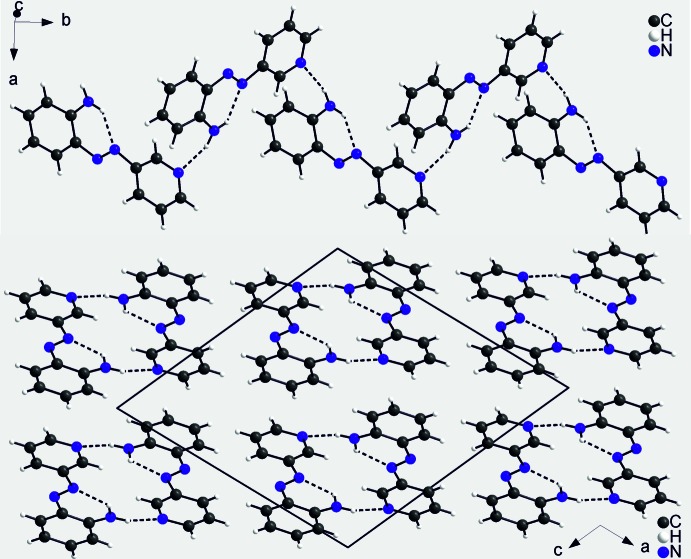
Crystal structure of the title compound showing a chain (top) and a view along the *b* axis (bottom). Intra- and inter­molecular hydrogen bonds are indicated by dashed lines.

**Figure 3:**
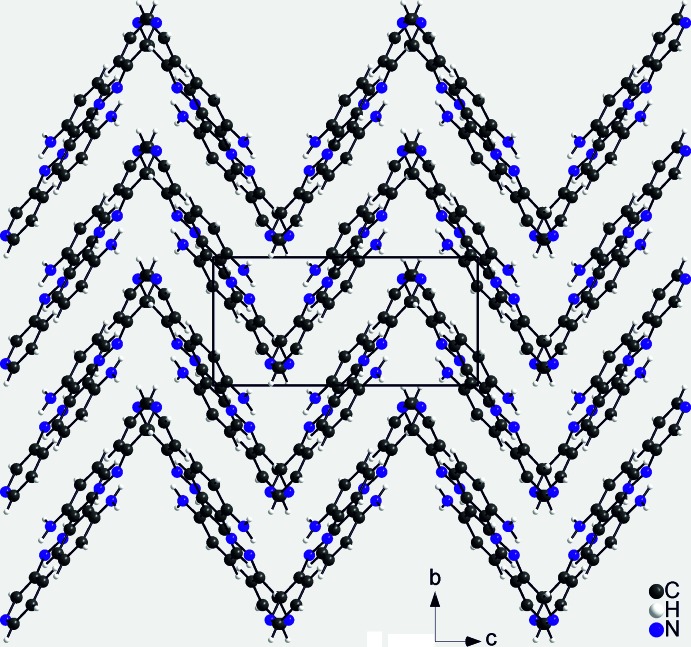
Crystal structure of the title compound in a view along the *a* axis.

In the crystal structure of the title compound, the mol­ecules are linked into chains along the *b*-axis direction *via* N—H⋯N hydrogen bonds between the amino hydrogen atom that is not involved in intra­molecular hydrogen bonding and one of the nitro­gen atoms of the azo group (Fig. 2 ▸, top). The N⋯H distance amounts to 2.163 (16) Å and the N—H⋯N angle of 159.7.14 (12)° is slightly bent, indicating that this is a relatively strong inter­action (Table 1 ▸). The dihedral angle between the pyridine ring that carries the acceptor N atom and the amino­phenyl moiety of a neighbouring mol­ecule that carries the donor group is 66.12 (8)°. Therefore, the mol­ecules exhibit a helical arrangement along the chain (Fig. 2 ▸, top). The chains are closely packed in such a way that each chain is surrounded by eight neighboring chains (Fig. 2 ▸, bottom). The mol­ecules exhibit a herringbone-like pattern along the *a* axis (Fig. 3 ▸) in which the pyridine and benzene rings of adjacent mol­ecules are perfectly coplanar. The distance between the ring planes is 3.462 Å and the centroid–centroid distance is 3.8040 (7) Å, indicating π–π inter­actions between the chains.

## Database survey   

According to a search of the Cambridge Structural Database (CSD; Groom *et al.*, 2016 ▸), 3-azo­pyridine mol­ecules substituted with an amino group in the *ortho*-position are unknown. However, one *ortho*-substituted phenyl-azo­pyridine was reported, *viz*. 2,6-di­amino-3-[(2-carb­oxy­meth­yl)phenyl­azo]pyridine (Tan *et al.*, 2010 ▸). 4-Amino­phenyl-azo­pyridines such as *N,N*-diethyl-4-[(*E*)-(pyridine-3-yl)diazen­yl]aniline (Draguta *et al.*, 2015 ▸) and *N*,*N*-dimethyl-4-(pyridine-3-yldiazen­yl)aniline (Draguta *et al.*, 2013 ▸) are also known. Furthermore, structure reports on 2-azo­pyridine mol­ecules that are substituted in the *ortho*-position, such as 5-[(5-bromo-2-pyrid­yl)azo]-2,4-toluenedi­amine (Jinzi *et al.*, 1984 ▸) and 5-[(3,5-di­bromo-2-pyrid­yl)azo]-2,4-di­amino­toluene (Kailiang *et al.*, 1985 ▸), have been published. Other azo compounds, substituted similarly to the title mol­ecule, are highly important for coordination chemistry, which is shown in their crystal structures (Maiti *et al.*, 2001 ▸, 2003 ▸; Pratihar *et al.*, 2005 ▸, 2007 ▸).

## Synthesis and crystallization   

The synthesis of the title compound can be performed in two steps.

**(i) Synthesis of 3-(2-acetanilide)azopyridine:** 2-nitro­acetanilide (4.00 g, 29.0 mmol) was dissolved in ethanol (150 ml). An aqueous solution of ammonium chloride (2.01 g, 37.7 mmol in 15 ml) was added. The mixture was warmed up to 313 K until the dispersion changed into a clear solution. After cooling to room temperature, zinc dust (4.93 g, 75.3 mmol) was added and the mixture was stirred for 1 h at 333 K. After filtration, the filtrate was poured into an aqueous ice-cooled iron(III) chloride solution (hexa­aqua complex, 5.40 g, 20.3 mmol in 150 ml) whereby a green solid precipitated. After 15 min of stirring, the solid was filtered off and washed with water. The crude product was a mixture of 2-nitro­soacetanilide and starting material, which was used for azo condensation without further purification. 3-Amino­pyridine (800 mg, 8.51 mmol) was dissolved in a mixture of pyridine (25 ml) and aqueous sodium hydroxide (5 ml, 25%). The crude product of 2-nitro­soacetanilide dissolved in pyridine (30 ml) was added to the solution containing the 3-amino­pyridine. The reaction mixture was stirred for 1 h at 353 K and overnight at room temperature. After addition of di­chloro­methane (200 ml) the phases were separated. The organic layer was washed with water twice and dried over sodium sulfate. The solvent was removed under reduced pressure. The crude product was purified by column chromatography (ethyl ester/*n*-hexane, *R*_f_ = 0.16). The product was obtained as an orange solid. Yield: 200 mg (0.83 mmol, 10%).

^1^H NMR (500 MHz, 300 K, CDCl3): δ = 9.88 (*s*, br, 1H, N-H), 9.11 (*d*, ^4^ *J* = 2.4 Hz, 1H), 8.71 (*dd*, ^3^ *J* = 4.7 Hz, ^4^ *J* = 1.5 Hz), 8.66 (*d*, *br*, ^3^ *J* = 8.3 Hz, 1H), 8.07 (*ddd*, ^3^ *J* = 8.2 Hz, ^4^ *J* = 2.4 Hz, ^4^ *J* = 1.5 Hz, 1H), 7.83 (*dd*, ^3^ *J* = 8.1 Hz, ^4^ *J* = 1.4 Hz 1H), 7.51–7.44 (*m*, 2H), 7.16 (*td*, ^3^ *J* = 8.1 Hz, ^4^ *J* = 1.3 Hz 1H), 2.26 (*s*, 3H) ppm. ^13^C NMR (150 MHz, 300 K, CDCl3): δ = 168.3, 151.9, 147.7, 146.3, 138.9, 136.4, 133.8, 127.3, 124.1, 123.4, 120.6, 120.4, 25.2 ppm. HRMS (EI): *m*/*z* [*M*]^+^ calculated for C_13_H_12_N_4_O: 240.10111, found: 240.10136.

**(ii) Synthesis of 2-[2-(pyridin-3-yl)diazen-1-yl]aniline:** 3-(2-acetanilide)azo­pyridine (640 mg, 2.66 mmol) was dissolved in methanol (50 ml). A sodium hydroxide solution (5 ml, 30%) was added and stirred for 6 h at 343 K. 2-[2-(Pyridin-3-yl)diazen-1-yl]aniline precipitated and was filtered off. The solid was washed with water. Recrystallization from acetone gave orange single crystals. Yield: 520 mg (2.63 mmol, 99%).

^1^H NMR (500 MHz, 300 K, CDCl_3_): *δ* = 9.08 (*dd*, ^4^ *J* = 2.4 Hz, ^5^ *J* = 0.8Hz, 1H), 8.63 (*dd*, ^3^ *J* = 4.7 Hz, ^4^ *J* = 1.6 Hz, 1H), 8.08 (*ddd*, ^3^ *J* = 8.2 Hz, ^4^ *J* = 2.4 Hz, ^4^ *J* = 1.6 Hz, 1H), 7.84 (*dd*, ^3^ *J* = 8.1 Hz, ^4^ *J* =1.4 Hz, 1H), 7.41 (*ddd*, ^3^ *J* =8.2 Hz, ^3^ *J* = 4.7 Hz, ^5^ *J* = 0.8 Hz, 1H),7.24 (*ddd*, ^3^ *J* = 8.3 Hz, ^3^ *J* = 7.1 Hz, ^4^ *J* = 1.4 Hz, 1H), 6.83 (*ddd*, ^3^ *J* = 8.1 Hz, ^3^ *J* = 7.1 Hz, ^4^ *J* = 1.3 Hz, 1H), 6.77 (*dd*, ^3^ *J* = 8.3 Hz, ^4^ *J* = 1.3 Hz, 1H), 6.01 (*s*, *br*, 2H, N—H) ppm. ^13^C NMR (150 MHz, 300 K, CDCl_3_): *δ* = 150.6, 148.3, 146.51, 143.0, 137.1, 133.1, 128.5, 126.4, 123.9, 117.5, 117.1 ppm. MS (EI, TOF): *m*/*z* (%) = 199 [*M*]^+^, 198 [*M*], 120 [*M* − C_5_H_4_N]^+^, 92 [*M* − C_6_H_6_N_2_]^+^.

Comparison of the experimental X-ray powder diffraction pattern with that calculated from single crystal data proves that the title compound was obtained as a pure phase (see Fig. S1 in the supporting information). The UV–Vis spectrum shows the strong π→π* band of the *trans* conformation (Fig. S2 in the supporting information). If the sample is exposed to light of 365 nm, no isomerization into the cis conformer is observed, and the sample starts to decompose. However, conversion of the amino to an amide group will probably restore the photochromic properties.

## Refinement   

Crystal data, data collection and structure refinement details are summarized in Table 2 ▸. The C—H hydrogen atoms were located in a difference map but were positioned with idealized geometry and refined with *U*_iso_(H) = 1.2*U*_eq_(C,N) using a riding model with C_aromatic_—H = 0.95 Å. The N—H hydrogen atoms were located in a difference map and were freely refined.

## Supplementary Material

Crystal structure: contains datablock(s) I. DOI: 10.1107/S2056989018008605/wm5450sup1.cif

Structure factors: contains datablock(s) I. DOI: 10.1107/S2056989018008605/wm5450Isup2.hkl

Fig. S1. Experimental (top) and calculated (bottom) X-ray powder diffraction patterns for the title compound, measured with copper radiation.. DOI: 10.1107/S2056989018008605/wm5450sup3.tif

Fig. S2. UV-Vis spectra for the title compound.. DOI: 10.1107/S2056989018008605/wm5450sup4.tif

Supporting information file. DOI: 10.1107/S2056989018008605/wm5450Isup5.cml

CCDC reference: 1848833

Additional supporting information:  crystallographic information; 3D view; checkCIF report

## Figures and Tables

**Table 1:** Hydrogen-bond geometry (Å, °)

*D*—H⋯*A*	*D*—H	H⋯*A*	*D*⋯*A*	*D*—H⋯*A*
N4—H1*N*4⋯N2	0.881 (16)	2.066 (15)	2.6922 (14)	127.3 (12)
N4—H2*N*4⋯N1^i^	0.904 (16)	2.163 (16)	3.0274 (14)	159.7 (14)

Symmetry code: (i) 
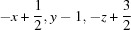
.

**Table 2:** Experimental details

Crystal data	
Chemical formula	C_11_H_10_N_4_
*M*_r_	198.23
Crystal system, space group	Monoclinic, *P*2/*n*
Temperature (K)	200
*a*, *b*, *c* (Å)	13.2798 (8), 5.9792 (3), 13.4130 (9)
β (°)	113.046 (7)
*V* (Å^3^)	980.03 (11)
*Z*	4
Radiation type	Mo *K*α
μ (mm^−1^)	0.09
Crystal size (mm)	0.20 × 0.15 × 0.15

Data collection	
Diffractometer	Stoe IPDS1
No. of measured, independent and observed [*I* > 2σ(*I*)] reflections	8233, 2137, 1731
*R*_int_	0.073
(sin θ/λ)_max_ (Å^−1^)	0.639

Refinement	
*R*[*F* ^2^ > 2σ(*F* ^2^)], *wR*(*F* ^2^), *S*	0.039, 0.113, 1.04
No. of reflections	2137
No. of parameters	145
H-atom treatment	H atoms treated by a mixture of independent and constrained refinement
Δρ_max_, Δρ_min_ (e Å^−3^)	0.21, −0.18

Computer programs: *X-AREA* (Stoe, 2008 ▸), *SHELXS97* and *XP* (Sheldrick, 2008 ▸), *SHELXL2014* (Sheldrick, 2015 ▸), *DIAMOND* (Brandenburg, 2014 ▸) and *publCIF* (Westrip, 2010 ▸).

## supplementary crystallographic information

### Crystal data

**Table:**

C_11_H_10_N_4_	*F*(000) = 416
*M**_r_* = 198.23	*D*_x_ = 1.344 Mg m^−^^3^
Monoclinic, *P*2/*n*	Mo *K*α radiation, λ = 0.71073 Å
*a* = 13.2798 (8) Å	Cell parameters from 8233 reflections
*b* = 5.9792 (3) Å	θ = 3.3–27.0°
*c* = 13.4130 (9) Å	µ = 0.09 mm^−^^1^
β = 113.046 (7)°	*T* = 200 K
*V* = 980.03 (11) Å^3^	Block, orange
*Z* = 4	0.20 × 0.15 × 0.15 mm

### Data collection

**Table:**

Stoe IPDS-1 diffractometer	*R*_int_ = 0.073
Phi scans	θ_max_ = 27.0°, θ_min_ = 3.3°
8233 measured reflections	*h* = −16→16
2137 independent reflections	*k* = −7→7
1731 reflections with *I* > 2σ(*I*)	*l* = −17→17

### Refinement

**Table:**

Refinement on *F*^2^	Hydrogen site location: mixed
Least-squares matrix: full	H atoms treated by a mixture of independent and constrained refinement
*R*[*F*^2^ > 2σ(*F*^2^)] = 0.039	*w* = 1/[σ^2^(*F*_o_^2^) + (0.0675*P*)^2^ + 0.0778*P*] where *P* = (*F*_o_^2^ + 2*F*_c_^2^)/3
*wR*(*F*^2^) = 0.113	(Δ/σ)_max_ < 0.001
*S* = 1.04	Δρ_max_ = 0.21 e Å^−^^3^
2137 reflections	Δρ_min_ = −0.18 e Å^−^^3^
145 parameters	Extinction correction: SHELXL2014 (Sheldrick, 2015), Fc^*^=kFc[1+0.001xFc^2^λ^3^/sin(2θ)]^-1/4^
0 restraints	Extinction coefficient: 0.054 (14)

### Special details

**Table:**

Geometry. All esds (except the esd in the dihedral angle between two l.s. planes) are estimated using the full covariance matrix. The cell esds are taken into account individually in the estimation of esds in distances, angles and torsion angles; correlations between esds in cell parameters are only used when they are defined by crystal symmetry. An approximate (isotropic) treatment of cell esds is used for estimating esds involving l.s. planes.

### Fractional atomic coordinates and isotropic or equivalent isotropic displacement parameters (Å^2^)

**Table:**

	*x*	*y*	*z*	*U*_iso_*/*U*_eq_	
C1	0.42109 (8)	0.49909 (17)	0.67373 (8)	0.0197 (3)	
C2	0.40530 (8)	0.65338 (19)	0.74372 (9)	0.0240 (3)	
H2	0.3449	0.6313	0.7638	0.029*	
N1	0.46934 (7)	0.83068 (16)	0.78456 (8)	0.0264 (3)	
C3	0.55368 (9)	0.85966 (19)	0.75476 (9)	0.0252 (3)	
H3	0.5997	0.9861	0.7817	0.030*	
C4	0.57689 (9)	0.71309 (19)	0.68624 (9)	0.0261 (3)	
H4	0.6382	0.7387	0.6680	0.031*	
C5	0.51034 (9)	0.53033 (18)	0.64487 (9)	0.0236 (3)	
H5	0.5248	0.4281	0.5979	0.028*	
N2	0.34292 (7)	0.32432 (14)	0.63669 (7)	0.0218 (2)	
N3	0.35904 (7)	0.19490 (15)	0.56895 (7)	0.0216 (2)	
C6	0.28594 (8)	0.01793 (17)	0.52757 (8)	0.0205 (3)	
C7	0.19205 (8)	−0.03083 (18)	0.55077 (8)	0.0221 (3)	
C8	0.13128 (9)	−0.22461 (19)	0.50278 (9)	0.0272 (3)	
H8	0.0687	−0.2617	0.5171	0.033*	
C9	0.16052 (9)	−0.3602 (2)	0.43605 (9)	0.0294 (3)	
H9	0.1186	−0.4903	0.4059	0.035*	
C10	0.25156 (10)	−0.30942 (19)	0.41168 (9)	0.0291 (3)	
H10	0.2705	−0.4022	0.3642	0.035*	
C11	0.31265 (9)	−0.12381 (19)	0.45757 (9)	0.0247 (3)	
H11	0.3747	−0.0897	0.4418	0.030*	
N4	0.15968 (9)	0.09982 (18)	0.61468 (9)	0.0308 (3)	
H1N4	0.2024 (12)	0.210 (2)	0.6510 (12)	0.034 (4)*	
H2N4	0.1081 (13)	0.045 (3)	0.6365 (13)	0.045 (4)*	

### Atomic displacement parameters (Å^2^)

**Table:**

	*U*^11^	*U*^22^	*U*^33^	*U*^12^	*U*^13^	*U*^23^
C1	0.0178 (5)	0.0203 (5)	0.0213 (5)	0.0009 (4)	0.0080 (4)	0.0027 (4)
C2	0.0186 (5)	0.0268 (6)	0.0293 (5)	0.0009 (4)	0.0125 (4)	−0.0011 (4)
N1	0.0232 (5)	0.0264 (5)	0.0313 (5)	0.0005 (4)	0.0125 (4)	−0.0045 (4)
C3	0.0219 (5)	0.0222 (5)	0.0309 (6)	−0.0024 (4)	0.0098 (4)	−0.0007 (4)
C4	0.0221 (5)	0.0284 (6)	0.0321 (6)	−0.0021 (4)	0.0152 (4)	0.0021 (5)
C5	0.0242 (5)	0.0251 (6)	0.0262 (6)	0.0005 (4)	0.0150 (4)	0.0000 (4)
N2	0.0211 (4)	0.0221 (5)	0.0240 (5)	−0.0010 (3)	0.0106 (4)	0.0000 (4)
N3	0.0220 (4)	0.0196 (5)	0.0238 (5)	−0.0001 (3)	0.0097 (4)	0.0019 (4)
C6	0.0203 (5)	0.0198 (5)	0.0211 (5)	0.0008 (4)	0.0078 (4)	0.0032 (4)
C7	0.0208 (5)	0.0240 (6)	0.0208 (5)	−0.0005 (4)	0.0073 (4)	0.0037 (4)
C8	0.0226 (5)	0.0300 (6)	0.0274 (6)	−0.0050 (4)	0.0080 (4)	0.0028 (5)
C9	0.0279 (6)	0.0238 (6)	0.0301 (6)	−0.0046 (4)	0.0045 (5)	−0.0016 (5)
C10	0.0304 (6)	0.0255 (6)	0.0296 (6)	0.0034 (5)	0.0099 (5)	−0.0029 (5)
C11	0.0236 (5)	0.0249 (6)	0.0267 (6)	0.0025 (4)	0.0110 (4)	0.0023 (4)
N4	0.0291 (5)	0.0351 (6)	0.0362 (6)	−0.0100 (4)	0.0214 (4)	−0.0073 (5)

### Geometric parameters (Å, º)

**Table:**

C1—C2	1.3895 (15)	C6—C11	1.4094 (15)
C1—C5	1.3959 (14)	C6—C7	1.4283 (14)
C1—N2	1.4186 (13)	C7—N4	1.3484 (15)
C2—N1	1.3350 (14)	C7—C8	1.4148 (15)
C2—H2	0.9500	C8—C9	1.3716 (17)
N1—C3	1.3397 (13)	C8—H8	0.9500
C3—C4	1.3892 (15)	C9—C10	1.4035 (17)
C3—H3	0.9500	C9—H9	0.9500
C4—C5	1.3781 (15)	C10—C11	1.3703 (16)
C4—H4	0.9500	C10—H10	0.9500
C5—H5	0.9500	C11—H11	0.9500
N2—N3	1.2738 (12)	N4—H1N4	0.881 (16)
N3—C6	1.3958 (13)	N4—H2N4	0.904 (16)
			
C2—C1—C5	117.88 (10)	C11—C6—C7	119.42 (10)
C2—C1—N2	116.26 (9)	N4—C7—C8	119.78 (10)
C5—C1—N2	125.86 (10)	N4—C7—C6	122.88 (10)
N1—C2—C1	124.34 (10)	C8—C7—C6	117.34 (10)
N1—C2—H2	117.8	C9—C8—C7	121.61 (10)
C1—C2—H2	117.8	C9—C8—H8	119.2
C2—N1—C3	116.94 (9)	C7—C8—H8	119.2
N1—C3—C4	122.94 (10)	C8—C9—C10	120.92 (10)
N1—C3—H3	118.5	C8—C9—H9	119.5
C4—C3—H3	118.5	C10—C9—H9	119.5
C5—C4—C3	119.55 (10)	C11—C10—C9	118.89 (10)
C5—C4—H4	120.2	C11—C10—H10	120.6
C3—C4—H4	120.2	C9—C10—H10	120.6
C4—C5—C1	118.35 (10)	C10—C11—C6	121.81 (10)
C4—C5—H5	120.8	C10—C11—H11	119.1
C1—C5—H5	120.8	C6—C11—H11	119.1
N3—N2—C1	113.14 (9)	C7—N4—H1N4	119.2 (9)
N2—N3—C6	117.34 (9)	C7—N4—H2N4	117.8 (10)
N3—C6—C11	113.80 (9)	H1N4—N4—H2N4	119.8 (14)
N3—C6—C7	126.78 (10)		
			
C5—C1—C2—N1	0.57 (16)	N3—C6—C7—N4	2.90 (17)
N2—C1—C2—N1	−178.53 (10)	C11—C6—C7—N4	−178.27 (10)
C1—C2—N1—C3	0.34 (16)	N3—C6—C7—C8	−177.68 (9)
C2—N1—C3—C4	−1.12 (16)	C11—C6—C7—C8	1.15 (15)
N1—C3—C4—C5	0.96 (17)	N4—C7—C8—C9	179.05 (10)
C3—C4—C5—C1	0.01 (16)	C6—C7—C8—C9	−0.39 (15)
C2—C1—C5—C4	−0.72 (15)	C7—C8—C9—C10	−0.88 (17)
N2—C1—C5—C4	178.28 (10)	C8—C9—C10—C11	1.36 (17)
C2—C1—N2—N3	176.88 (9)	C9—C10—C11—C6	−0.56 (17)
C5—C1—N2—N3	−2.14 (15)	N3—C6—C11—C10	178.29 (9)
C1—N2—N3—C6	−179.80 (8)	C7—C6—C11—C10	−0.69 (16)
N2—N3—C6—C11	−176.92 (9)	C1—N2—N3—C6	−179.80 (8)
N2—N3—C6—C7	1.96 (15)		

### Hydrogen-bond geometry (Å, º)

**Table:**

*D*—H···*A*	*D*—H	H···*A*	*D*···*A*	*D*—H···*A*
N4—H1*N*4···N2	0.881 (16)	2.066 (15)	2.6922 (14)	127.3 (12)
N4—H2*N*4···N1^i^	0.904 (16)	2.163 (16)	3.0274 (14)	159.7 (14)

Symmetry code: (i) −*x*+1/2, *y*−1, −*z*+3/2.
